# NINJA-OPS: Fast Accurate Marker Gene Alignment Using Concatenated Ribosomes

**DOI:** 10.1371/journal.pcbi.1004658

**Published:** 2016-01-28

**Authors:** Gabriel A. Al-Ghalith, Emmanuel Montassier, Henry N. Ward, Dan Knights

**Affiliations:** 1 Biomedical Informatics and Computational Biology, University of Minnesota, Minneapolis, Minnesota, United States of America; 2 University of Nantes, Nantes, France; 3 Department of Computer Science and Engineering, University of Minnesota, Minneapolis, Minnesota, United States of America; 4 Lawrence University, Appleton, Wisconsin, United States of America; University of California Davis, UNITED STATES

## Abstract

The explosion of bioinformatics technologies in the form of next generation sequencing (NGS) has facilitated a massive influx of genomics data in the form of short reads. Short read mapping is therefore a fundamental component of next generation sequencing pipelines which routinely match these short reads against reference genomes for contig assembly. However, such techniques have seldom been applied to microbial marker gene sequencing studies, which have mostly relied on novel heuristic approaches. We propose NINJA Is Not Just Another OTU-Picking Solution (NINJA-OPS, or NINJA for short), a fast and highly accurate novel method enabling reference-based marker gene matching (picking Operational Taxonomic Units, or OTUs). NINJA takes advantage of the Burrows-Wheeler (BW) alignment using an artificial reference chromosome composed of concatenated reference sequences, the “concatesome,” as the BW input. Other features include automatic support for paired-end reads with arbitrary insert sizes. NINJA is also free and open source and implements several pre-filtering methods that elicit substantial speedup when coupled with existing tools. We applied NINJA to several published microbiome studies, obtaining accuracy similar to or better than previous reference-based OTU-picking methods while achieving an order of magnitude or more speedup and using a fraction of the memory footprint. NINJA is a complete pipeline that takes a FASTA-formatted input file and outputs a QIIME-formatted taxonomy-annotated BIOM file for an entire MiSeq run of human gut microbiome 16S genes in under 10 minutes on a dual-core laptop.

## Author Summary

The analysis of the microbial communities in and around us is a growing field of study, partly because of its major implications for human health, and partly because high-throughput DNA sequencing technology has only recently emerged to enable us to quantitatively study them. One of the most fundamental steps in analyzing these microbial communities is matching the microbial marker genes in environmental samples with existing databases to determine which microbes are present. The current techniques for doing this analysis are either slow or closed-source. We present an alternative technique that takes advantage of a high-speed Burrows-Wheeler alignment procedure combined with rapid filtering and parsing of the data to remove bottlenecks in the pipeline. We achieve an order-of-magnitude speedup over conventional techniques without sacrificing accuracy or memory use, and in some cases improving both significantly. Thus our method allows more biologists to process their own sequencing data without specialized computing resources, and it obtains more accurate and even optimal taxonomic annotation for their marker gene sequencing data.

This is a *PLOS Computational Biology* Methods paper

## Introduction

The advent of next-generation sequencing technologies, combined with major advances in molecular and bioinformatics techniques, have enabled rapid growth in the culture-independent sequencing of amplified marker genes (amplicons) from environmental microbial communities. The major benefit of amplicon sequencing is that it allows reasonable resolution of taxonomic composition in these communities at a fraction of the cost of deep metagenomic sequencing. Once these sequences are generated, a common analysis approach is to bin them by sequence identity into operational taxonomic units (OTUs)[1–4]. For environments containing a large fraction of novel taxa, one must rely on unsupervised (“de novo”) clustering of amplicons to convert the raw reads to features representing organisms belonging to distinct evolutionary clades. On the other hand, in habitats with mostly well-characterized microbes, we have the option of matching the generated amplicon sequences to reference databases containing example marker genes from known taxa [5]. A hybrid approach may also be used, where sequences are first compared to a reference database, with subsequent de novo clustering of those that failed to match. As the number of published culture-independent amplicon-based surveys of microbial communities continues to grow, our ability to rely on reference sequences also increases. However, although the crucial analysis step of mapping generated amplicons to reference marker genes has received much attention from the microbial bioinformatics field, with a variety of solutions proposed [6–10], there is much room for improvement in terms of speed, accuracy, memory footprint, and openness of code. NINJA-OPS, our portable, open-source OTU picking pipeline, realizes these goals.

Originally conceived as a means to make data more compressible, the Burrows-Wheeler transform (BWT) [11] is a lossless, reversible transformation that effectively positions series of like characters close to each other in a way that can easily be undone to recover the original data. It involves creating a circular suffix array, sorting the final column lexicographically, and storing that column as the transformed data for later compression. This algorithm also has the interesting property of enabling rapid substring search, with O(1) order of growth in finding exact string matches. As long as there is an efficient indexing scheme that stores the indices of the transformed bases into the original string, the BWT can be used for fast database substring search amounting to binary searching (or looking up via rank matrix) the transformed reference string representation and mapping back to the original, and has hence been employed in a number of commonly used DNA alignment tools [11–14]. Although these tools are approximate methods due to the high additional computational cost of performing optimal local or global alignment search when mismatches occur, they are generally fast and widely used in the genome-enabled research community (http://bowtie-bio.sourceforge.net/bowtie2/other_tools.shtml).

Here we demonstrate that BWT-enabled DNA alignment can be effectively used for accurate and fast assignment of marker-gene sequences to a reference database. We present the NINJA-OPS pipeline utilizing several novel contributions to achieve an order of magnitude speedup and higher accuracy when compared to commonly used approaches (or up to two orders of magnitude when combined with denoising). To test the accuracy and efficacy of our approach, we perform closed-reference OTU-picking on a wide range of biological data sets from varied environments.

Accuracy was evaluated using an optimal aligner which produces a BLAST-style %ID for each query sequence against the reference sequence chosen by the OTU picking method. Speed was assessed as the elapsed time from parsing the correctly-formatted input FASTA file, which is accelerated by NINJA’s fast C parser and simple format requirements, until the alignment (against a pre-generated database) has terminated. However, it may be useful to note that NINJA also significantly speeds up the subsequent steps of tallying reads, incorporating taxonomic annotations, and producing an OTU table in sparse BIOM 1.0 format, as well as other steps prior to the alignment such as reverse complementing and trimming reads. Hence, the NINJA pipeline accelerates many stages of the OTU-picking pipeline in addition to the alignment step.

## Methods

### Pipeline overview

**Fig 1:**
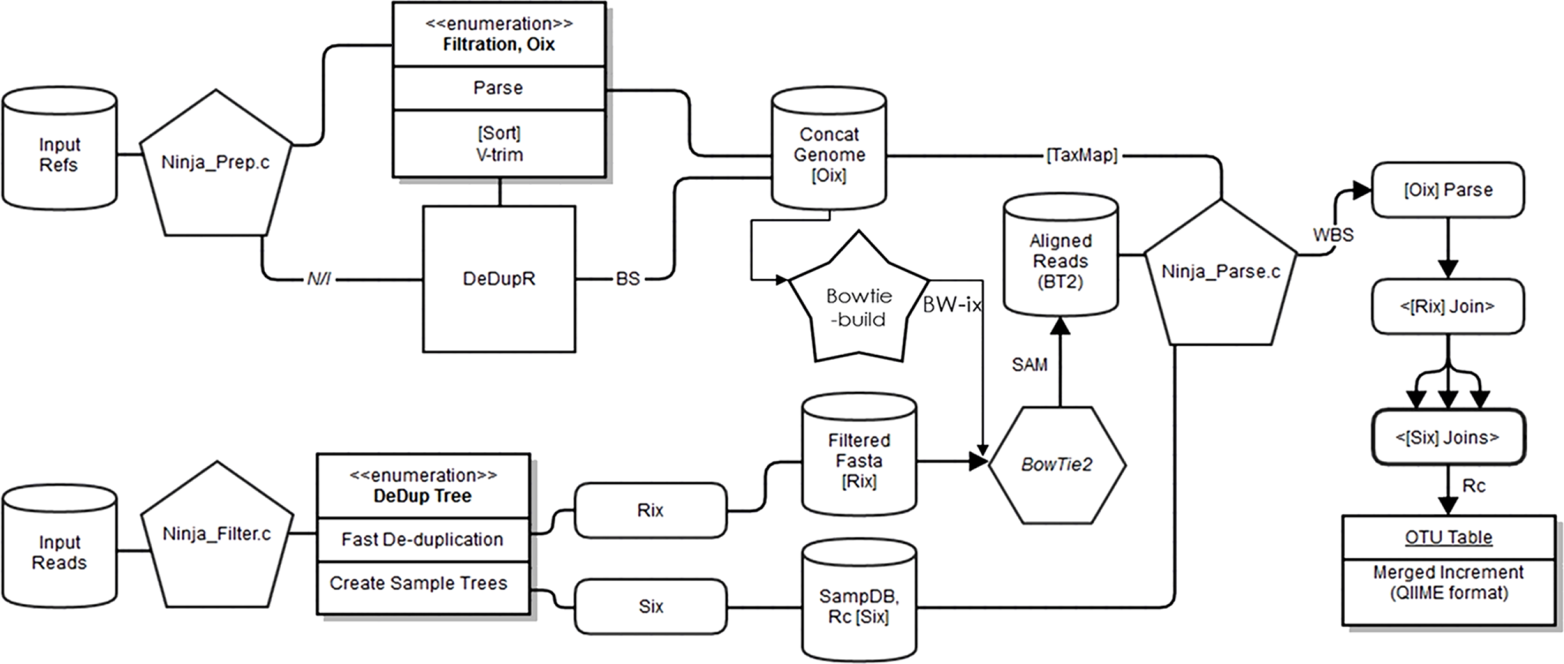
Schematic of the NINJA pipeline. NINJA core programs are represented by pentagons, data files by cylinders, processes within a program as lists, index operations as rounded rectangles, and other swappable programs by other shapes. The entire upper-left branch of the schematic (from input references to bowtie-build and TaxMap) does not need to be performed if using an existing database, such as that supplied with NINJA. The python wrapper encompasses the remaining two branches (bottom and right) for convenience. In general, Ninja_prep prepares the concatesome, Ninja_filter prepares the reads for alignment, bowtie2 (or any BWT-enabled aligner) performs the alignment, and Ninja_parse merges the various pieces into a complete OTU table.

The pipeline follows three stages: filtering, aligning, and parsing. After forming the concatenated reference string, called the “concatesome,” from the individual references, NINJA applies a powerful filtering step which uses a 3-way radix quicksort on string pointers to rapidly de-duplicate millions of reads, construct a sample dictionary, and output a reduced-size filtered FASTA file and sample dictionary (Fig 1). The program implements this lossless filtering approach as well as a lossy variant, making use of singleton filtering as well as statistical profiling over the entire set of reads to exclude reads with a user-defined number of duplicates or rare segments (k-mers) appearing below a user-defined threshold of prevalence. The lossy filtering, which is not enabled by default, is intended to identify reads with probable read error independently, and speeds up the resulting alignment by excluding such reads from the BWT aligner. This adds an additional speedup because BWT string matching spends a disproportionate amount of search effort to align erroneous or low-identity reads. Because choice of k (from 8 to 14) and prevalence threshold are highly domain- and dataset-specific, it is difficult to issue a general recommendation for this setting. Although we have found k = 8 to k = 14 at a threshold of 0.05%-0.01% to be a safe minimum for 16S data, user experimentation is recommended.

The NINJA filter step also performs reverse-complementing and sequence length trimming at the same time as the other filtering steps. Because of this simultaneous multi-step filtering, no intermediate files are created prior to the alignment stage, and all filtering steps are performed rapidly in optimized C code on data structures already in memory. This takes a fraction of the time used by other filtering pipelines which perform sequential operations often written in general-purpose scripting languages and generate numerous large intermediate files after each step. Using the base NINJA filter parameters, the entire filtering process itself takes approximately 10–20% of the time it takes to align the resulting filtered file when using all optional filtering steps.

Next, the filtered reads are aligned against a reference database containing the (the concatesome) via any BWT-derived short read aligner such as BWA[11], Bowtie[13]/Bowtie2[12], hpg-aligner [15], SOAP2 [16]—or, more broadly, any read aligner whatsoever capable of outputting to headerless SAM format[17] and suppressing unmatched input reads. Utilizing SAM is much faster than BAM (binary compressed SAM) after deduplication, as the alignment step is not I/O bound and the overhead of BAM’s additional compression/decompression step can be significant. We have chosen to standardize NINJA around Bowtie2 for our tests and publish the command line options for Bowtie2 as we have found it to be suitable for the purposes of BLAST-identity-based OTU picking. Following alignment, the resulting SAM file is fed to the NINJA parsing step, which takes in the sample dictionary metadata as well as an optional taxonomy map to rapidly re-assign each de-duplicated read to the biological sample(s) in which it originally occurred, add taxonomy annotation to each picked OTU, bin all reads by their matched OTU into a sample-by-OTU matrix (OTU table), and output the result in sparse BIOM 1.0 format or a tab-delimited human-readable legacy QIIME format. This can also be incorporated into an open-reference OTU-picking pipeline.

### Burrows-Wheeler transform

The BWT has received a lot of attention in the alignment of short reads to a reference genome, and now enjoys routine use in clinical and other settings as a well-vetted technique for mapping short DNA reads to a longer reference, where it is known as Burrows-Wheeler alignment. The BWT is based on the principle that a long string of text can be reversibly transformed to reduce the complexity of substring queries to effectively two binary searches into the transformed representation of the original string, which is then converted back to indices into the original reference string with a short walk-back (the BW Last-First, or LF walk) or lookup. The efficacy of this approach in matching short reads to a reference database of numerous short reference marker genes has remained largely unexplored [1].

#### Forming the concatesome

We concatenate the reference gene sequences to form a single long string. Although this synthetic chromosome is highly repetitive, the BWT effectively enables alignment against all identical reference prefixes simultaneously, which are narrowed down as alignment progresses and differences (or errors) accrue. This enables us to leverage the advantages of the BW technique for amplicon matching and evaluate its quality and speed compared to some of the current heuristic techniques[7][18][19] that have emerged specifically to address the marker-gene matching problem in microbial metagenomics. This step also exists in varying forms and efficiencies in recent versions of some BWT-based read aligners. It is performed by NINJA in a rapid and unified way, enabling drop-in replacement of any BWT-based algorithm, by joining reference sequences them into a single long sequence and recording the location of each OTU in the new longer sequence. The concatesome is output as a new single-line FASTA file, and the original indices are output as an index table of OTU names and IDs. The concatesome serves as the reference for the BWT-enabled aligner to construct its database. For bowtie2, which we have used as the pipeline’s BWT aligner for the subsequent analyses, this is accomplished with the bowtie-build command.

#### K-mer-based denoising

NINJA’s filter step implements a variant of 3-way radix quicksort [20] that achieves high binning and de-duplication performance on amplicon datasets using minimal RAM. This is due to its general high performance on strings combined with a median pivot scheme and increasing performance on data with larger numbers of duplicates, which is especially true for amplicon datasets. Trimming is implemented in *C* and is performed before deduplication during the parsing process by halting the read pointer and skipping to the next sequence, speeding up the parse and using less memory. Trimming at this stage can also speed up the subsequent deduplication by feeding shorter sequences to the sorting algorithm. Additionally, reads that are uniformly trimmed (preferably informed by average quality score prior to running NINJA) are more likely to have identical matches, resulting in a more condensed deduplicated output file and faster subsequent alignment. Reverse complementing is performed after any trimming at the time of output file writing by populating the write buffer in reverse with the result of a lookup performed on each sequential character in the read.

The k-mer-based lossy filtering step is performed by incrementing an array of counters indexed by the integer representation of each *k*-mer formed as the read head slides across each base in the read. Formally, each *k*-mer is represented as a binary string with 2*k* bits constructed as follows:

$$x_{2i,2i+1}=\left\{ \begin{array}{c} A:00\\ C:01\\ G:10\\ T:11 \end{array}\right.$$

The array of counters is of length 2^2*k*^, and contains the number of instances observed of a given *k*-mer in the query DNA sequences. We use *k* = 8 by default (adjustable at compile-time with a practical maximum of 14), giving a counter array of length 2^16^, occupying up to 2^16^ × 64 bits or 4 MB of RAM.

The counter is incremented per k-mer per read to represent the number of that k-mer’s duplicates in the dataset. The resulting count array after all sequences are parsed forms the empirical *k*-mer frequency distribution across all input reads. For amplicon data, the sorted frequency distribution resembles an exponential curve, with a small number of *k*-mers well represented and others with fewer occurrences (and many with none, depending on k-mer size). With sufficient number of input reads, the likelihood of observing a k-mer of a given rareness can be estimated from the area under the empirical frequency distribution. By checking the number of occurrences of a given k-mer against the empirical duplicate count at that probability threshold, we can determine if a given k-mer is an outlier and hence discard the read. The user can modify the stringency of outlier calling by setting a different observation frequency cutoff. We have found empirically that k-mers (with k = 8 to k = 14) appearing less often than 0.5%-0.1% (parameter D 0.0001 when calling the underlying ninja_filter executable) provide a reasonably safe threshold for low-strength 16S amplicon denoising, but the user is encouraged to evaluate various thresholds in context of the sequencing technology used, as well as read depth, community diversity, and experimental biases.

#### Singleton-based denoising

The k-mer-based filtering provides a highly flexible and sensitive approach to denoising, but we have also implemented a very simple denoising step, which is to exclude all singleton (or doubleton, etc.) sequences from the alignment step. This is based on the premise that any sequences that appears twice or more is highly unlikely to have been the result of sequencing error, while sequences that appear exactly once are likely erroneous or so rare that they are just at the detection threshold. In several data sets we have found that this eliminates approximately 10% of the total sequences due to high likelihood that they contain errors. The resulting OTU assignments are generally unchanged when excluding singletons (S1 Fig), and yet the runtime achieves an additional 3-10x speedup over the non-denoised approach. We recommend these settings (parameter “-d 2”) for most users unless they have very low read counts.

#### Alignment

We have conducted most of our testing using bowtie2 as the BWT-enabled aligner for NINJA. To perform OTU-picking in a manner consistent with current tools, a custom set of command-line parameters was used. We sought to maintain concordance with various USEARCH operating assumptions which have become the standard for marker gene sequencing (as used in the QIIME pipeline). The primary criterion used for matching should be percent identity, which weights all matches in an alignment 1, and mismatches of any type 0. The sum of all such scores across the alignment, spanning the length of the query sequence, is divided by the total length of that alignment. Matches to ambiguous bases are also not penalized, nor are sequential gaps weighted any differently than single gaps (no affine penalty). Further, once a reasonable match has been identified in the database matching these criteria, the search terminates. The following options for bowtie2 target this behavior:

--np0--mp"1,1"--rdg"0,1"--rfg"0,1"--score-min"L,0,-0.03"-k1--norc

The percent ID is specified by setting the third argument to the minimum scoring function (—score-min) as %ID/100–1. In this case, to match with 97% ID, the parameter is set to 97/100–1 = -0.03. It is recommended that users not modify other parameters of bowtie2 pertaining to scoring criteria or output format, to maintain compatibility with the %ID match criterion and downstream NINJA parsing. It is further recommended to use the included python wrapper so that all steps from reading the formatted FASTA to OTU table creation are performed automatically for the user with a single command.

Additionally, for compatibility with NINJA and to save space, reads that fail to align are suppressed and no headers are printed to the output SAM file and the concatesome built with bowtie2-build is used as the reference database. The input sequences are the result of the filtering step. Full examples of the bowtie2 command are given in the online documentation. By default, we include presets for fast or very sensitive matching. Very sensitive matching typically improves the quality of matches noticeably at the expense of a 3-6x longer running time.

#### Parsing

The final step in NINJA-OPS combines parsing the alignments, assigning OTU identity and taxonomy, and tallying OTU counts in each sample as an output OTU table (in BIOM 1.0 or **legacy** QIIME format). This is performed by NINJA-OPS using the information provided in sample dictionary generated by the filtering step, as well as the alignment data produced by the aligner and (optionally) a user-supplied table of OTU taxonomic assignments (which is also provided pre-compiled with NINJA-OPS). This parsing step is I/O bound and runs in under a second on our test computer and dataset.

#### Comparison

To compare NINJA-OPS against USEARCH 8 (an earlier version of USEARCH is used in the QIIME pipeline [1]), we built the USEARCH database using the same multiple reference FASTA used to create the concatesome, the Greengenes 97% OTU representative sequence database. We performed single-strand alignment with the following fast USEARCH settings, which correspond to the stringency used for USEARCH in the QIIME pipeline:

-usearch_global input.fna-db db.udb -id0.97-blast6out hits.b6-strand plus -maxaccepts1-maxrejects32-threads1

## Results

Benchmarks for this article were performed on a 2013 MacBook Air (MacBook Air 6,2) with a dual-core Core i7 CPU and 256GB SSD.

### Preparation step

The runtime performance of the database generation is significantly longer than is practical to perform on the fly. This step only needs to be performed once for each reference database. Although ninja_prep performs the concatenation of references rapidly (it is I/O bound on the Macbook’s SSD), the BWT program may spend a long time generating the BW index. For bowtie2 on our test machine, this takes over half an hour (with a maximum of one thread) on the Greengenes 97% OTU representative sequence database. For this reason, it is best to store and use pre-compiled databases for all subsequent alignments, and NINJA-OPS is distributed with a number of pre-compiled databases for commonly performed 16S bacterial marker-gene OTU matching.

### Filter step

NINJA filtering takes approximately 10–15% of the alignment time. For our 1.6 million read 175bp test data, without additional processing, filtering runs in 3.5 seconds and outputs a de-duplicated FASTA file approximately 1/5 the size of the original.

### Alignment step

**Fig 2:**
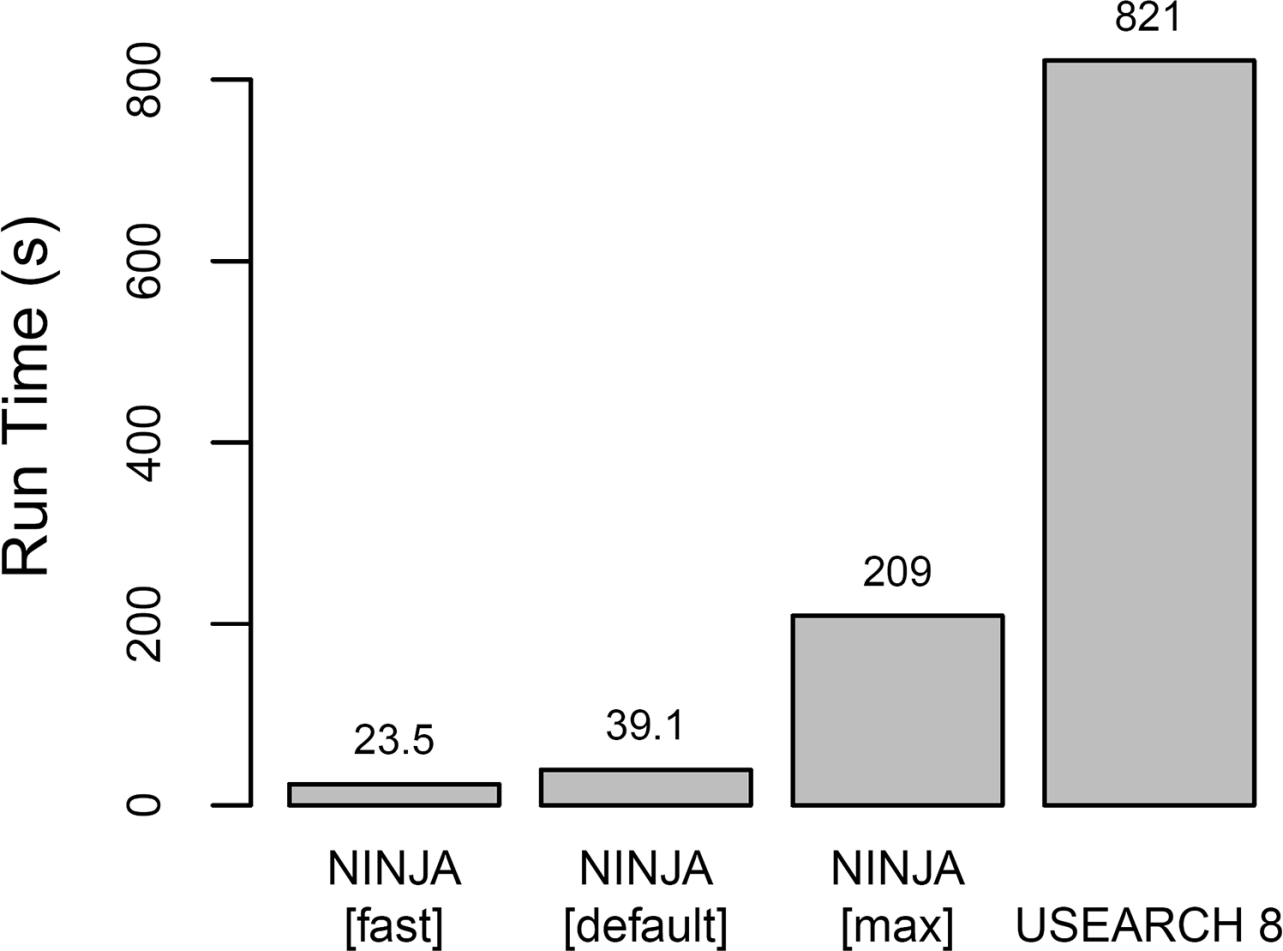
Benchmark of runtimes for NINJA compared to USEARCH 8 in a single-threaded environment. Multi-threaded alignments are faster. For NINJA only, this represents the entire time from parsing the initial FASTA file to the completion of the OTU table. The sortMeRNA program took substantially longer than USEARCH 8 (approx. 8000s; bar not shown to preserve scale).

Bowtie2 with the settings mentioned in methods aligns the entire test dataset of 1.6 million 175bp reads in under 40 seconds on a single thread of the test laptop. Performance for the default and maximum-fidelity (“max”) NINJA presets were measured (Fig 2). The “max” preset not only demonstrates higher accuracy than either the default preset or USEARCH, but also retains significantly more reads. The “fast” preset displays similar accuracy characteristics to “default,” but misses about 2% of the alignments detected by the latter, usually of the lowest identity. Total pipeline runtime on the same dataset decreases to less than 10 seconds when using the recommended singleton-based denoising option (parameter “D 2”), in combination with default preset. In addition, the speedup versus USEARCH 8 was measured using the default preset without denoising (“D 0”) across different datasets (S2 Fig). RAM usage during alignment was 205MB in all cases, while that of USEARCH 8 was 720MB. Using multiple threads during alignment decreases the running time further, but speedup is sublinear, having somewhat more advantage in datasets with longer reads or higher error rates (and hence more difficult alignments).

### Parse step

Parsing with ninja_parse takes roughly 0.2–3 seconds on datasets in the size range included here (0.5–2 million sequences). Outputting to legacy tab-delimited format instead of BIOM increases the runtime by a second or two. A Python-based convenience wrapper distributed with NINJA adds additional overhead if the user requests a fasta file containing the sequences that failed to match the database.

### Accuracy

**Fig 3:**
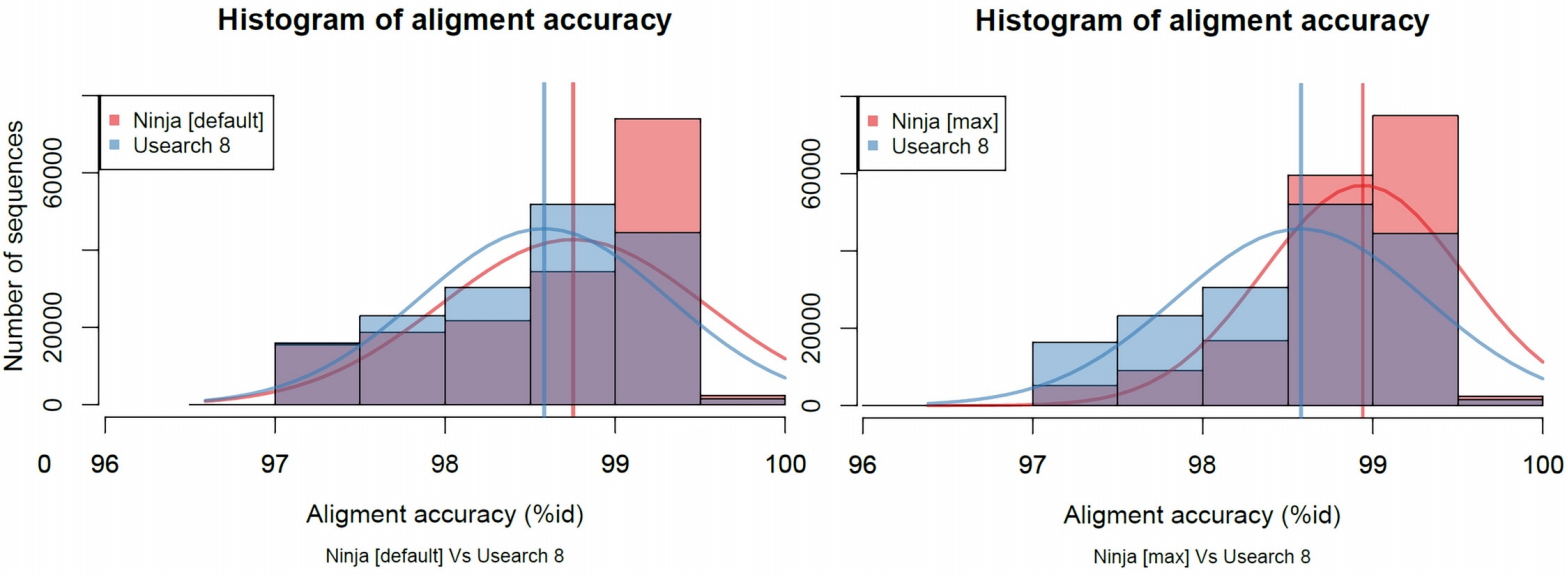
Comparison of NINJA and USEARCH 8 best match accuracy using all unique matches. These histograms show the distribution of matches by algorithm, where NINJA match proportion is in red, USEARCH in blue, and the overlap in purple. The right-most bars in each histogram show more red than purple, indicating NINJA aligned more reads at higher accuracy than USEARCH in the corresponding bins. The thick vertical bars correspond to the average alignment accuracy, and the thin curves represent the interpolated distribution. A Student’s t-test on the mean alignment accuracy shows NINJA’s mean ID was 98.9%, while USEARCH 8 produced 98.7% with p-value < 2.2e-16. For USEARCH 8, fast settings are used (corresponding to the defaults in the QIIME pipeline). Total number of unique sequences mapped are 168,761, 170,063, and 169,313, for NINJA [default], NINJA [max], and USEARCH 8, respectively.

**Fig 4:**
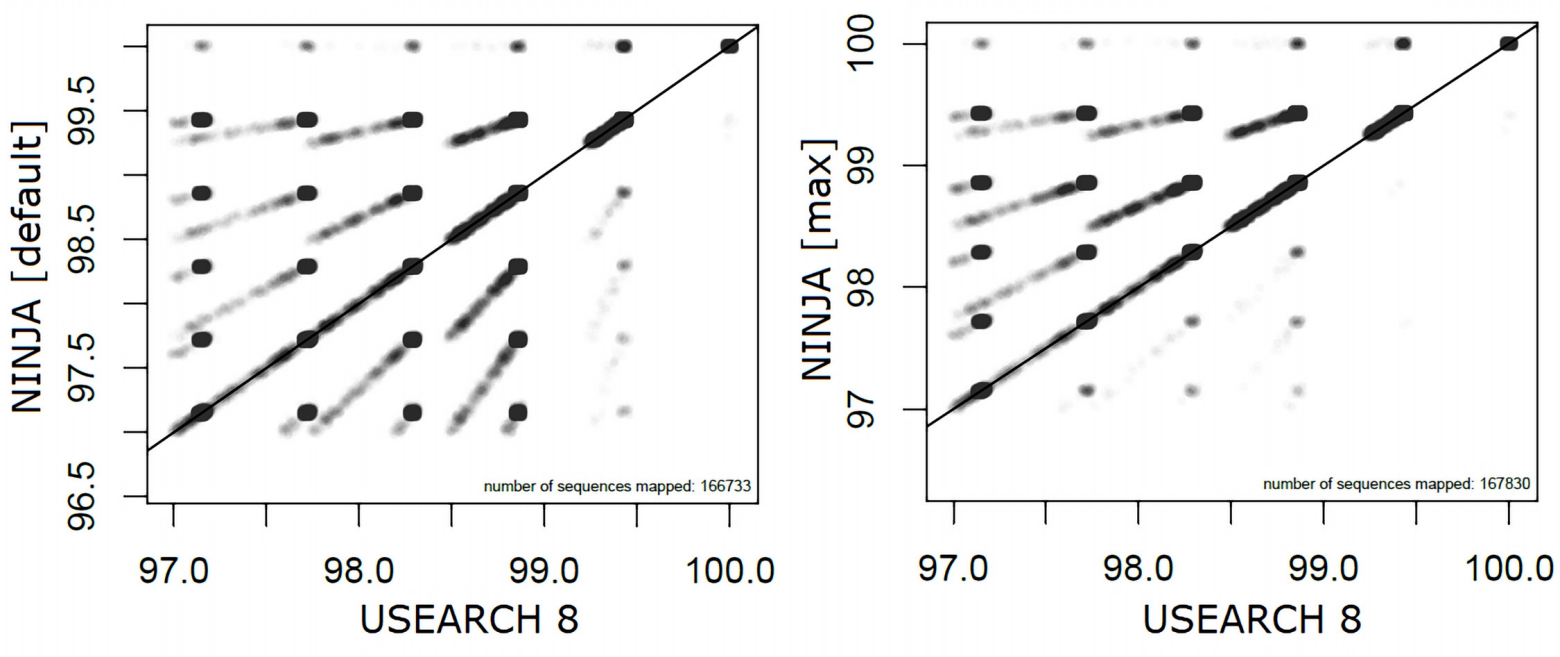
Alignment accuracy of NINJA vs USEARCH 8 (where both reported a match). Each point on the graph represents a sequence for which both tools found a valid alignment. A point’s position along the X axis corresponds to alignment score (in %ID) for the match chosen by USEARCH 8, and its position on the Y axis corresponds to the alignment score against the match chosen by NINJA. Points along the diagonal represent sequences for which both tools picked the same quality match. Points above the diagonal correspond to sequences for which NINJA produced more accurate hits, and points below the diagonal represent sequences for which USEARCH 8 produced more accurate hits. Note the presence of a line at the top of the graph showing a number of sequences for which NINJA selected a perfect match from the database while USEARCH 8 could not.

To assess the accuracy of the alignments found by NINJA, and to compare them to existing tools, we calculated the optimal alignment, using a semi-global version of the Smith-Waterman algorithm, of each query sequence with the reference sequence assigned by a given tool. We found that NINJA (default preset) generally finds higher-accuracy matches than USEARCH 8 (Mann-Whitney U test p < 2.2e-16) (Figs 3 and 4). In a published dataset containing healthy subjects and patients with Crohn’s disease the two methods produced the same list of differentiated genera across disease conditions with occasional disagreements about the direction of the association (S3 Fig). NINJA produced a comparable percentage of matches with default preset to USEARCH (S4 Fig), and generally comparable taxonomic assignments despite some interesting differences (S5 Fig).

## Discussion

**Fig 5:**
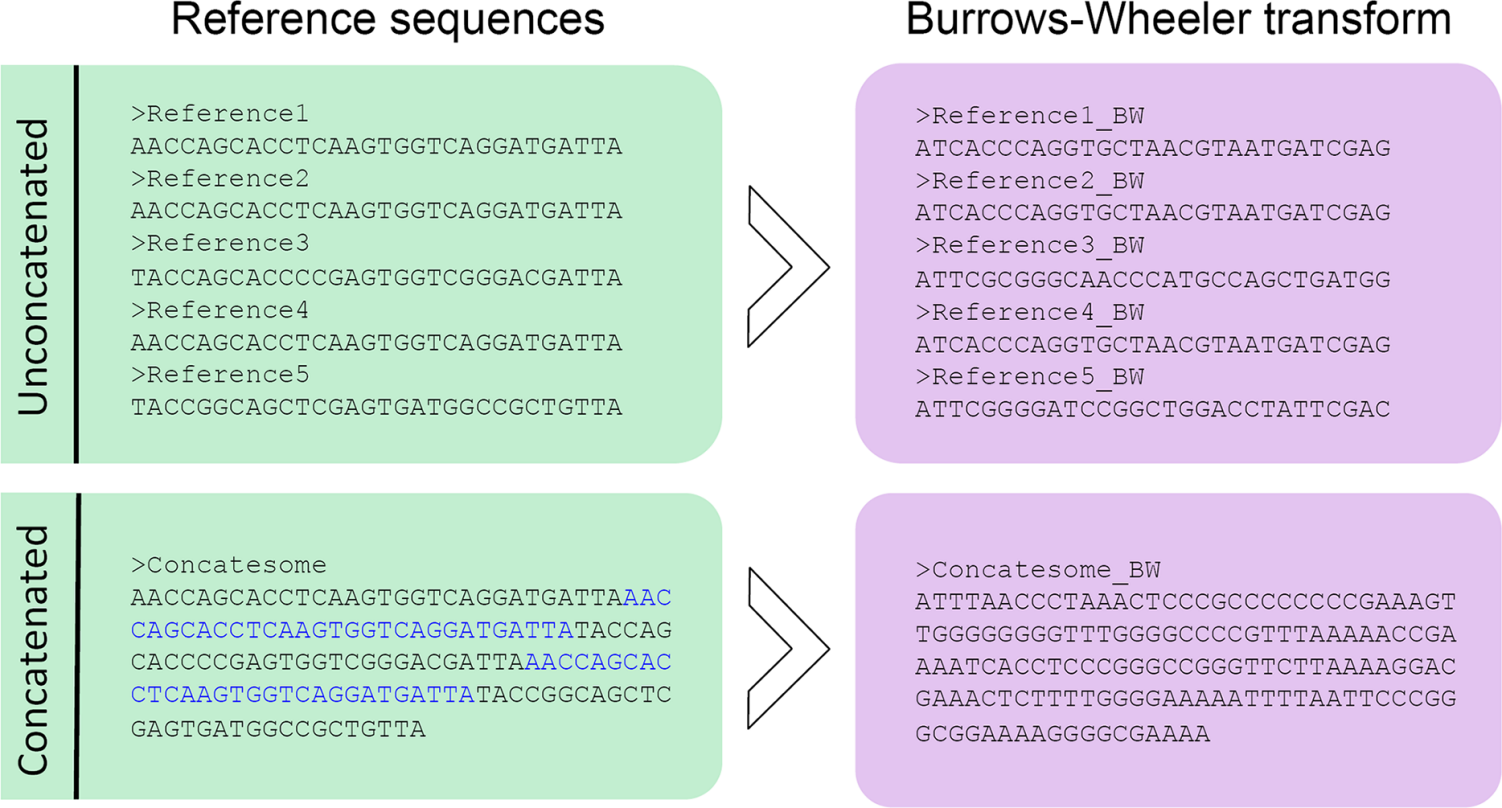
Burrows-Wheeler transform on concatenated sequences. Example of the Burrows-Wheeler transform on both small sequences (top) and a concatenated longer sequence (bottom). The concatenated sequence is more easily searchable using the LF walk. NINJA forms a BWT-compatible concatesome that can be used interchangeably among various BWT-based aligners as an artificial reference chromosome. This concatenated sequence serves as a reference against which environmentally-obtained marker sequences are aligned.

Our tool leverages a combination of several novel approaches to accomplish an order of magnitude speedup over existing methods without compromising accuracy, and in many cases NINJA-OPS is more accurate than popular existing tools. In combination with a recommended denoising step, the pipeline achieves up to two orders of magnitude speedup over USEARCH 8. The key innovation of this tool is our use of a single long reference genome, or concatesome, composed of concatenated marker genes. This approach allows NINJA to leverage the benefits of the Burrows-Wheeler transform (Fig 5). The code is available at http://ninja-ops.ninja (or https://github.com/GabeAl/NINJA-OPS).

### Deduplication and NINJA-filter

Optimizations within NINJA-OPS include tweaks to the parsing and filtering programs to increase the throughput of the processes leading up to the alignment. Deduplication is a viable strategy in marker-gene sequencing contexts because samples usually consist of fewer taxa than there are reads, and in fact are often dominated by a few highly abundant species. This results in a large number of identical reads which can be filtered out to reduce the alignment time. In human gut datasets which are quality-trimmed (or where the marker gene reads are of approximately equal length), this may result in losslessly discarding 80% of the reads as duplicates, depending on the microbial community sampled, which can speed up the downstream alignment step substantially (S6 Fig). A sequence-to-sample(s) dictionary keeps track of the abundance of each sequence in each sample to ensure that each original sequence is properly accounted for wherever it was originally found. By default, NINJA-filter also performs read compaction (parameter “-d 1"), which normalizes for variation in read lengths within a dataset by treating reads which are subsets of longer reads as copies of the longer reads. This increases consistency of OTU calling as well as decreasing runtime. This behavior can easily be disabled (parameter “-d 0”).

An optional beneficial feature during the filter step is the ability to perform lossy denoising. NINJA performs this in two ways. The first and most straightforward for amplicon reads is to discard singleton reads (parameter “-d 2”); that is, reads that have no identical match in the entire list of queries, or which are not perfectly contained in a longer read. This can be extended as the user desires from singletons to doublets and so on (parameter “-d 3”, “-d 4”, etc.). The second form of denoising is discarding reads judged to be erroneous by breaking each read into its component overlapping k-mers and comparing each of these k-mers to the counts of that k-mer in an empirical distribution of all k-mers in the body of input reads. Reads with k-mers that fail to meet user-defined criteria for support (appearing under a certain % in the dataset) are discarded completely from subsequent analysis. The resulting speedup for the downstream alignment is often much greater than the proportion of reads discarded, because Burrows-Wheeler alignment programs expend a disproportionately large amount of effort attempting to align erroneous reads that will not match the database compared to non-erroneous reads which will often find perfect (or near-perfect) matches in a well-populated database. The BW substring search is designed for perfect substring searches, so it performs most efficiently in aligning reads that have few to no mismatches with a subsequence of the database. This is also why NINJA and BWT tools perform most effectively when the alignment identity is high (%ID in the mid-to upper-90’s, with taxonomic resolutions at the level of genus or finer). Performance of BWT-based tools is expected to increase as the diversity of available reference sequences increases, because the probability of finding a perfect match likewise increases.

One early concern as we were considering how to most effectively construct the concatesome was that some reads would align by chance to the boundary between two concatenated marker genes, which would produce a meaningless mapping. However, in practice, such an occurrence is exceedingly unlikely to occur in end-to-end marker gene alignments at genus-level or greater resolution due to the high identity expected over the entire length of the input read. This is even more true of marker gene alignment, where reads are much more similar to each other than in shotgun data, and the possible sites of alignment seeding are likewise similar, with significantly less randomness than would produce alignments with the boundary region by chance. The prevalence of such reads in our 16S test data is accordingly less than 1 in 1,000,000 reads aligned. Furthermore, in the unlikely event that such an alignment does occur, it is trivial to discard it in the final parsing step by testing whether the index at the end of the alignment is equal to or greater than the starting index of the subsequent marker gene. NINJA-OPS automatically discards reads that map to junctions between concatenated marker genes.

An interesting finding that corroborates past findings [21] is that the commonly used bowtie, bowtie2, and BWA alignment tools do not scale linearly with increasing read length. However, due to the ability to substitute alternative BWT-based alignment programs for the alignment step, it is possible to use alternatively optimized variants such as HPG Aligner, which uses uncompressed suffix arrays instead of “traditional” BWT but shares many of the same characteristics with the added benefit of better scaling for longer reads. GPU-accelerated variants of the original algorithm are also available [22]. Additionally, NINJA-OPS is not restricted to the domain of 16S OTU picking, although it is distributed with a pre-built 16S database. Marker genes such as ITS for fungal identification [24], bacterial *rpoB* [25], and the recently proposed *Cpn*60 universal bacterial barcode [26] are easily incorporated into NINJA-OPS simply by compiling the included “ninja_prep.c” and running it on an appropriately-formatted FASTA file containing the desired marker sequences, followed by the BWT-based aligner’s database generation step. Further, NINJA-OPS can be incorporated as a preliminary step in another pipeline; for instance, NINJA-OPS can be used to group reads prior to de novo assembly [27]. This flexibility of the pipeline in allowing substitution of the aligner itself, as well as the marker gene database used, makes NINJA-OPS applicable for situations and optimizations beyond what were envisioned at the time of writing, and enable the pipeline to keep pace with emerging technologies in the sequencing and computing spheres alike.

## Supporting Information

S1 Fig Comparison of taxon abundance with singleton filtering (D2) vs no singleton filtering (D1) by taxonomic level. The plots show, from left to right, the scatterplot of log abundances of all matched taxa in a dataset of 6.5 million 225-base-pair sequences at progressively higher taxonomic specificity, along with best fit lines for each. The axes correspond to log abundance within the dataset, and each dot to an arbitrary taxon abundance in the singleton denoised (Y-axis) and non-denoised (X-axis) OTU tables. The left plot shows the family-level concordance (Pearson = 0.9901727, Spearman = 0.9848349), the middle shows genus-level concordance (Pearson = 0.9845869, Spearman = 0.974338), and the right shows species-level concordance (Pearson = 0.9789319, Spearman = 0.9604182).
(TIF)

S2 Fig The amount of speedup relative to USEARCH 8 using NINJA’s default preset without denoising. The speedup varies by dataset. Note that the Gevers dataset [23], for which the default NINJA preset is over 21 times faster, is fairly representative of human gut communities.
(TIF)

S3 Fig Significantly expressed taxa in an IBD dataset (q-value < 0.05). Top left: shows concordance between NINJA and QIIME 1.8 (USEARCH/UClust). This is the most typical case. Top right: diverging trends between groups. Despite being significantly different across groups, directionality of the trend is inverted between Control and IC/UC groups for the two methods. Bottom left: preservation of general trends but difference in taxonomic abundance of [Ruminococcus]. Bottom right: NINJA reports significance difference in [Eubacterium] expression while QIIME+USEARCH/Uclust does not.
(TIF)

S4 Fig Proportion of successful database matching. The percentage of reads NINJA (default preset, no denoising, “D 0”) successfully maps to the database are compared to USEARCH 8. There is very little difference in numbers of reads mapped to the database across datasets.
(TIF)

S5 Fig Comparison of genus-level taxonomic concordance between Ninja (X-axis) and USEARCH 8 (Y-axis). Each point represents a genus-level assignment, with coordinates along each axis corresponding to the total number of reads mapped to that particular genus by either Ninja (X-axis) or USEARCH 8 (Y-axis). Distance from the diagonal represents discordance of taxon calls.
(TIF)

S6 Fig Speedup of the alignment step after filtering (without denoising). Filtering can provide upwards of 2.5x improvement in alignment performance, depending on the dataset. Smaller or more unique datasets see more modest improvements. Datasets with communities of redundant sequences benefit the most. This benchmark was performed without denoising or read compaction (parameter “D 0”). Using NINJA-OPS with denoising or read compaction provides a substantially greater speedup.
(TIF)

S7 Fig QR code for the NINJA-OPS download website. This Quick Response (QR) code, stylized to resemble a gapped multiple alignment of DNA sequences (including clip art from public domain at http://www.wpclipart.com), is a functional QR code provided for convenient linking to the NINJA-OPS source code and pre-built binaries hosted at http://ninja-ops.ninja, and for convenient sharing and distribution of the tool in poster presentations, slide shows, and other visual presentations.
(TIF)
