# Supplementary figures and images for: NINJA-OPS: Fast Accurate Marker Gene Alignment Using Concatenated Ribosomes
Source: PLoS Comput Biol. 2016 Jan 28;12(1):e1004658. doi: 10.1371/journal.pcbi.1004658 (PMC4731464; doi:10.1371/journal.pcbi.1004658)

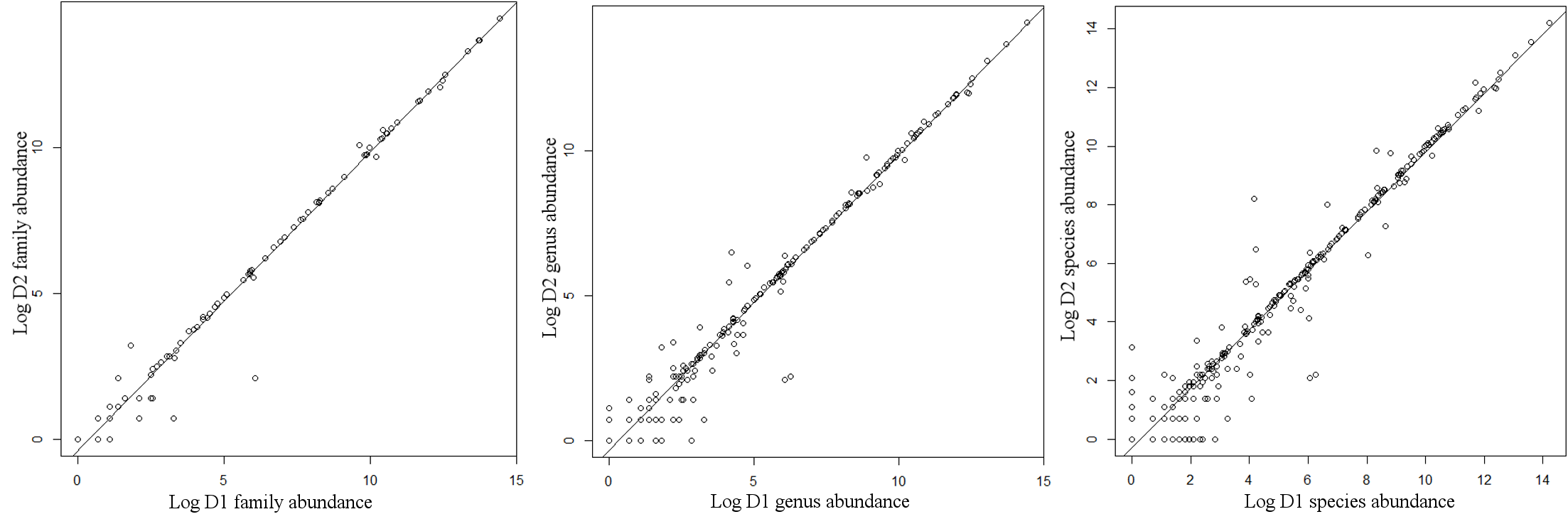

Supplement: S1 Fig — The plots show, from left to right, the scatterplot of log abundances of all matched taxa in a dataset of 6.5 million 225-base-pair sequences at progressively higher taxonomic specificity, along with best fit lines for each. The axes correspond to log abundance within the dataset, and each dot to an arbitrary taxon abundance in the singleton denoised (Y-axis) and non-denoised (X-axis) OTU tables. The left plot shows the family-level concordance (Pearson = 0.9901727, Spearman = 0.9848349), the middle shows genus-level concordance (Pearson = 0.9845869, Spearman = 0.974338), and the right shows species-level concordance (Pearson = 0.9789319, Spearman = 0.9604182). (TIF) [file pcbi.1004658.s001.tif]

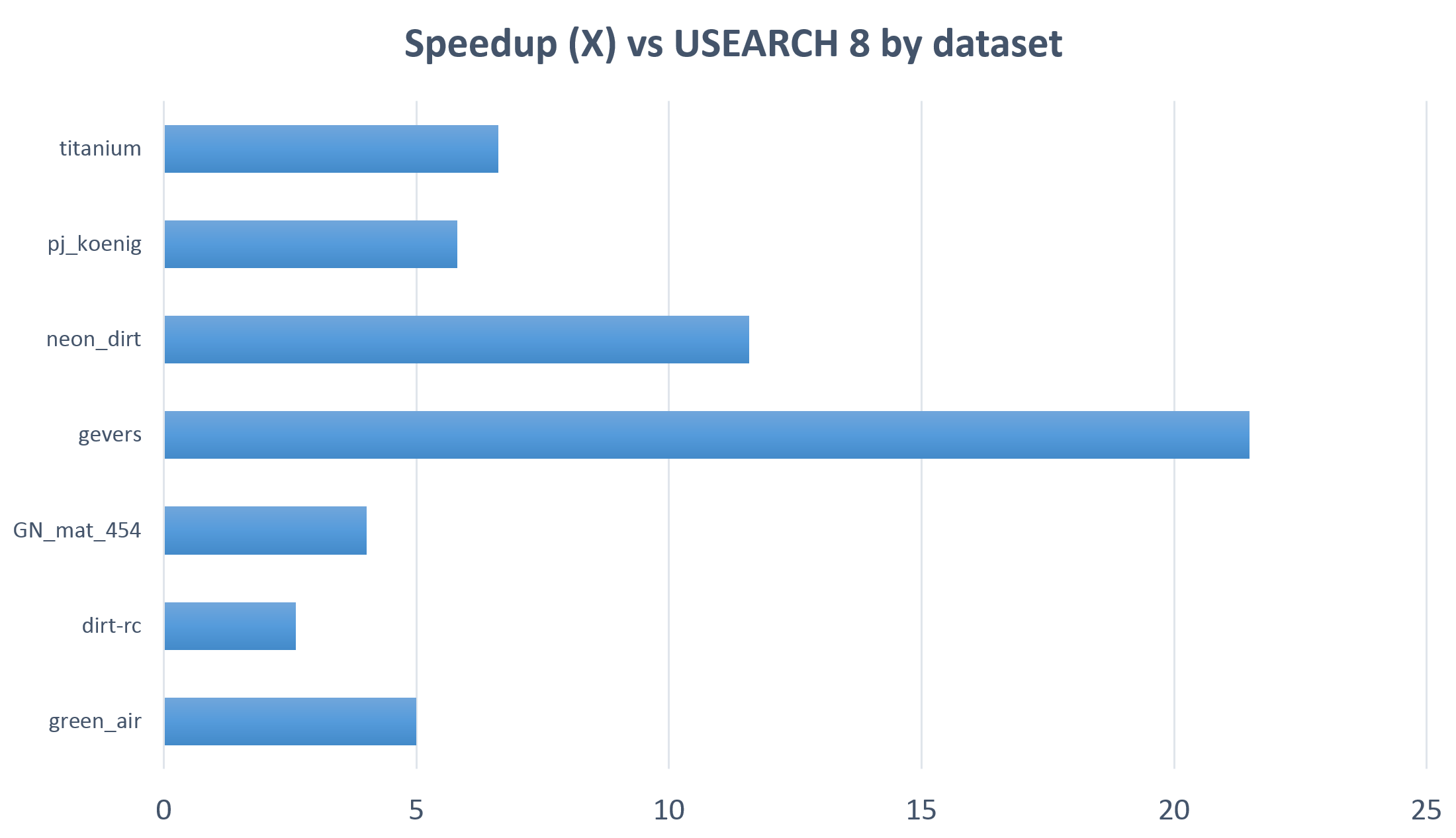

Supplement: S2 Fig — The speedup varies by dataset. Note that the Gevers dataset [23], for which the default NINJA preset is over 21 times faster, is fairly representative of human gut communities. (TIF) [file pcbi.1004658.s002.tif]

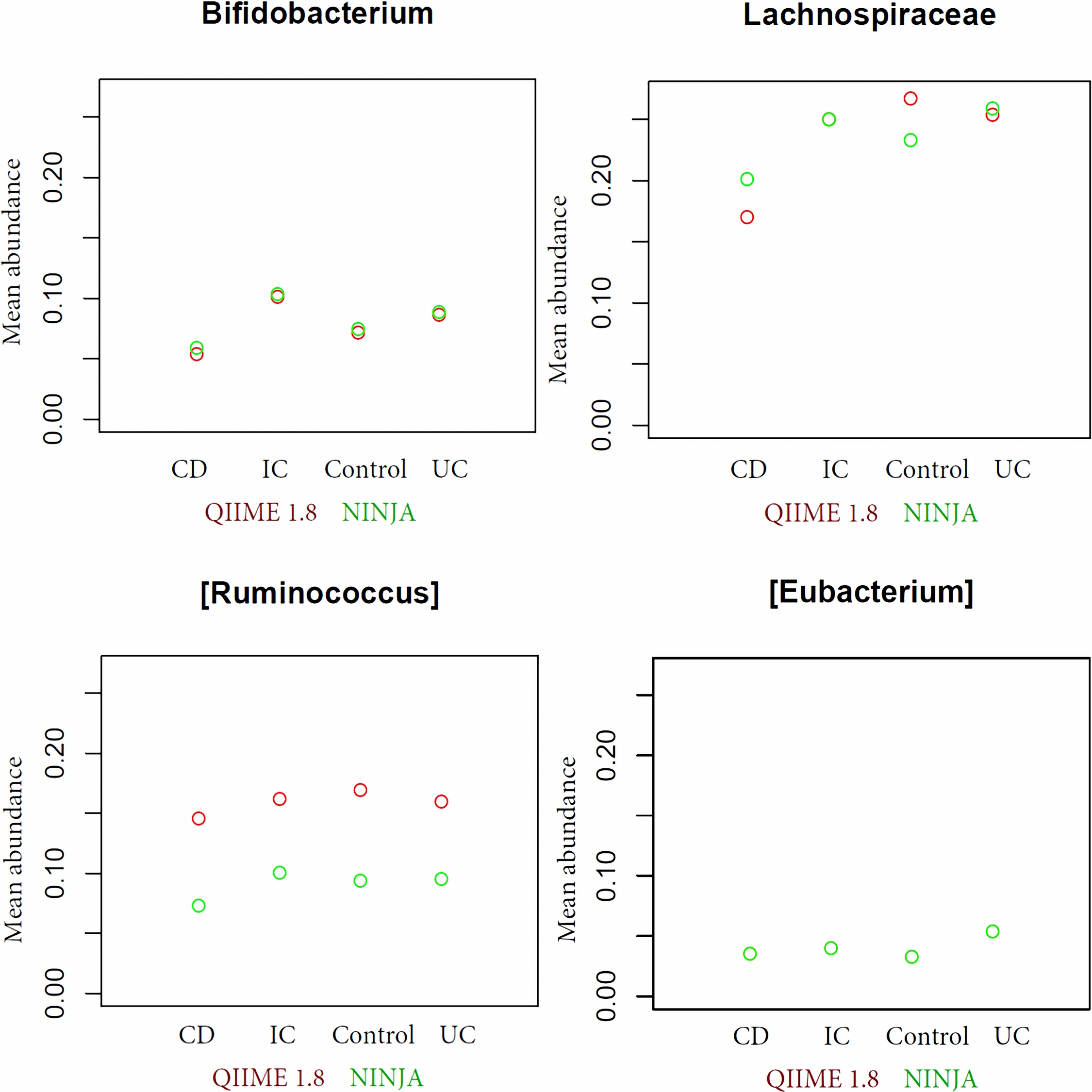

Supplement: S3 Fig — Top left: shows concordance between NINJA and QIIME 1.8 (USEARCH/UClust). This is the most typical case. Top right: diverging trends between groups. Despite being significantly different across groups, directionality of the trend is inverted between Control and IC/UC groups for the two methods. Bottom left: preservation of general trends but difference in taxonomic abundance of [Ruminococcus]. Bottom right: NINJA reports significance difference in [Eubacterium] expression while QIIME+USEARCH/Uclust does not. (TIF) [file pcbi.1004658.s003.tif]

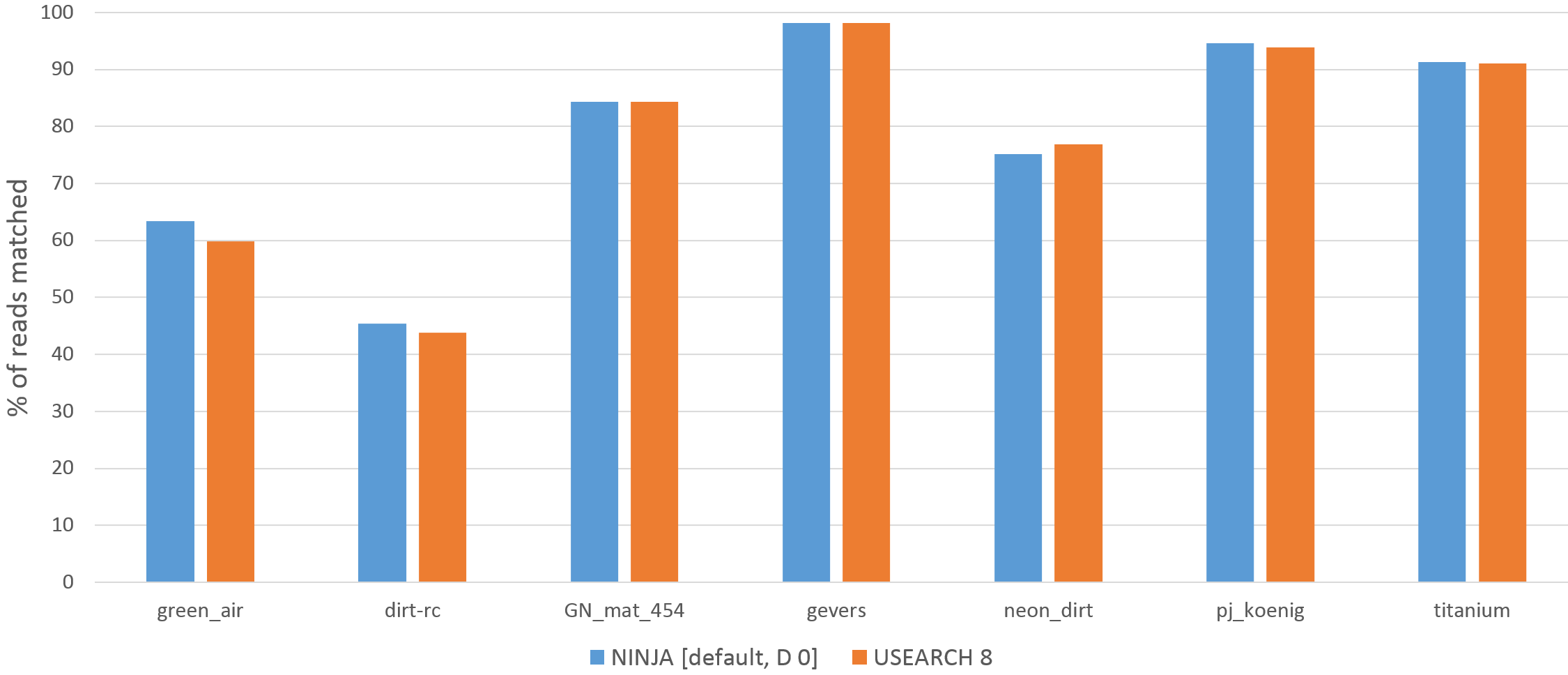

Supplement: S4 Fig — The percentage of reads NINJA (default preset, no denoising, “D 0”) successfully maps to the database are compared to USEARCH 8. There is very little difference in numbers of reads mapped to the database across datasets. (TIF) [file pcbi.1004658.s004.tif]

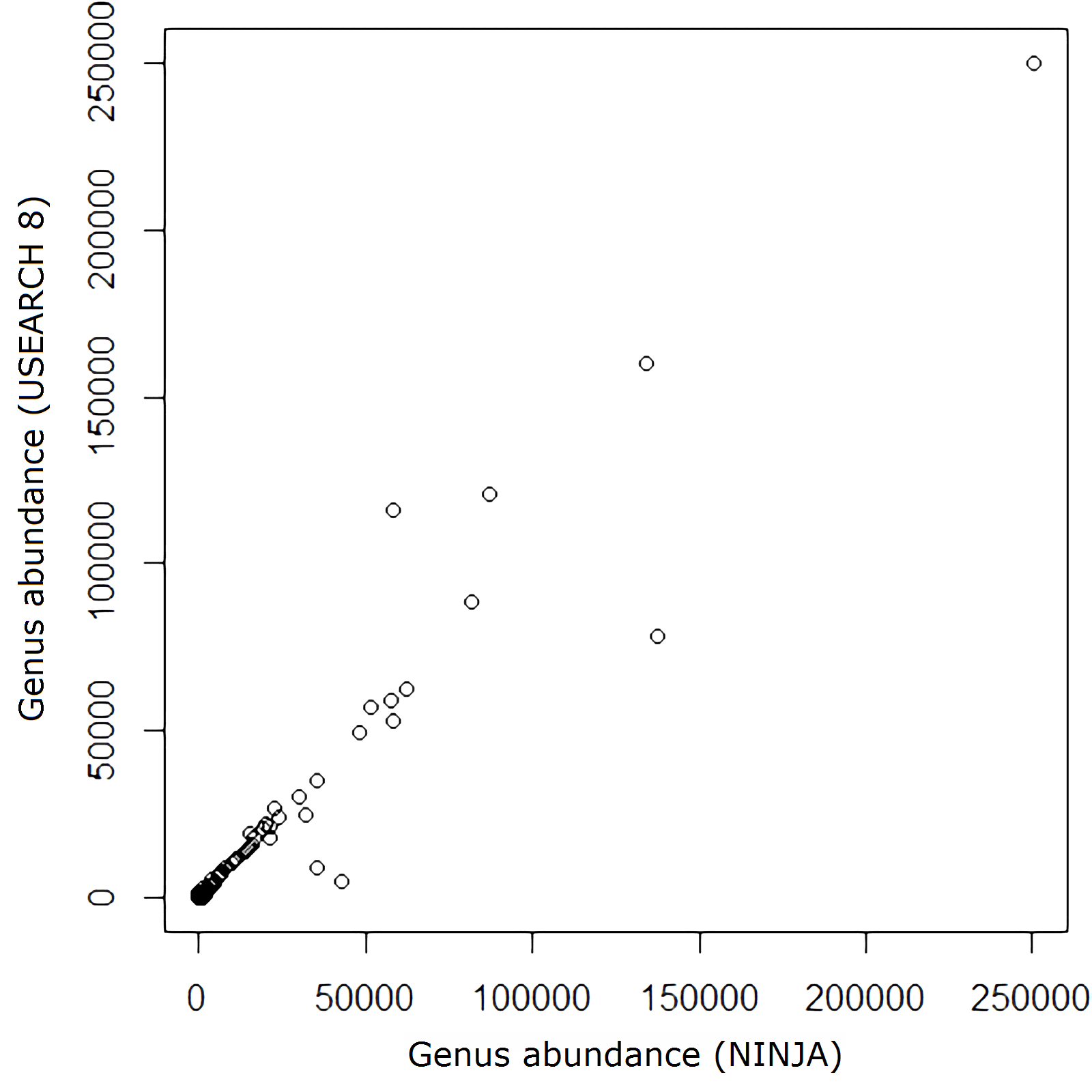

Supplement: S5 Fig — Each point represents a genus-level assignment, with coordinates along each axis corresponding to the total number of reads mapped to that particular genus by either Ninja (X-axis) or USEARCH 8 (Y-axis). Distance from the diagonal represents discordance of taxon calls. (TIF) [file pcbi.1004658.s005.tif]

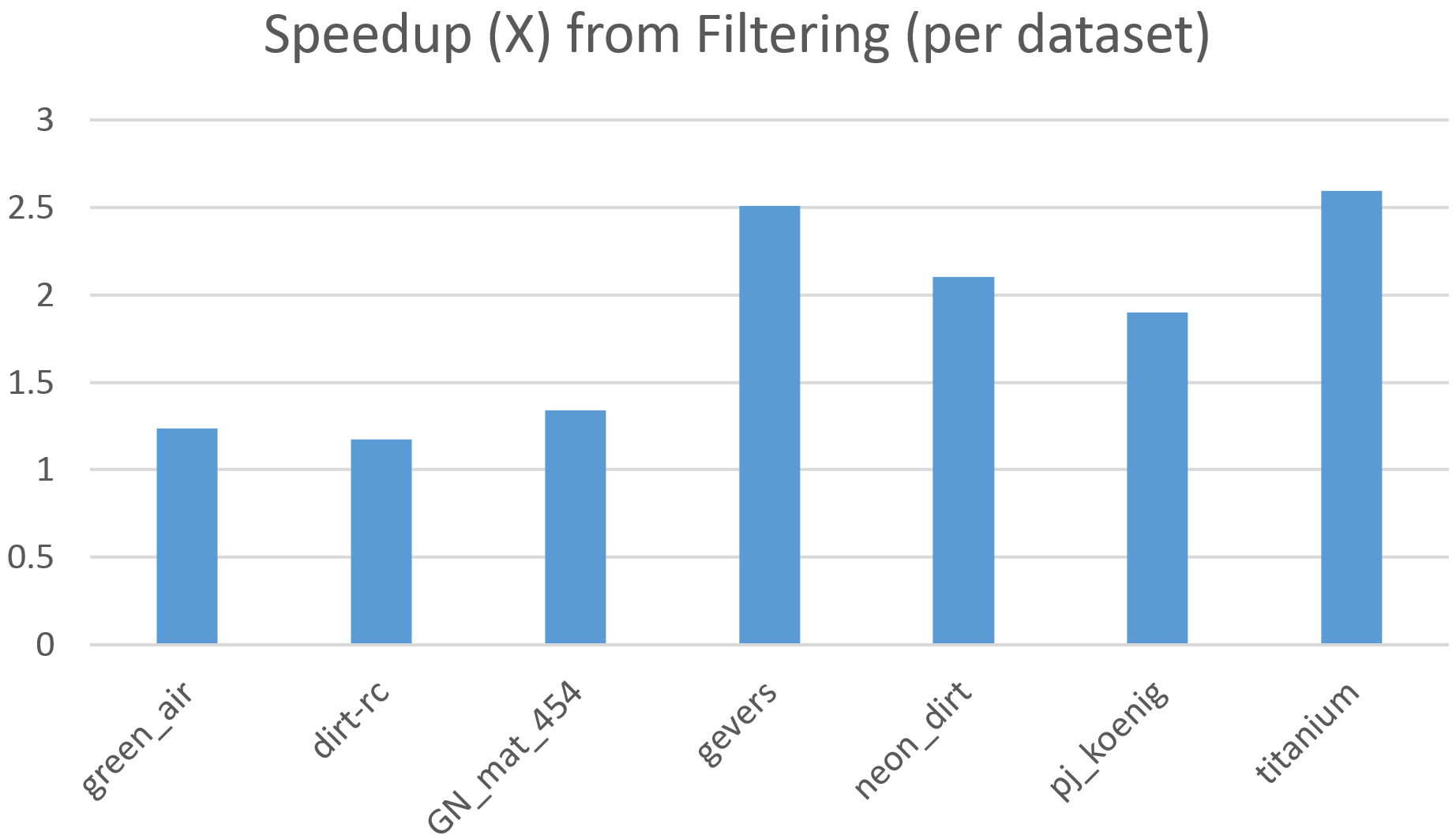

Supplement: S6 Fig — Filtering can provide upwards of 2.5x improvement in alignment performance, depending on the dataset. Smaller or more unique datasets see more modest improvements. Datasets with communities of redundant sequences benefit the most. This benchmark was performed without denoising or read compaction (parameter “D 0”). Using NINJA-OPS with denoising or read compaction provides a substantially greater speedup. (TIF) [file pcbi.1004658.s006.tif]

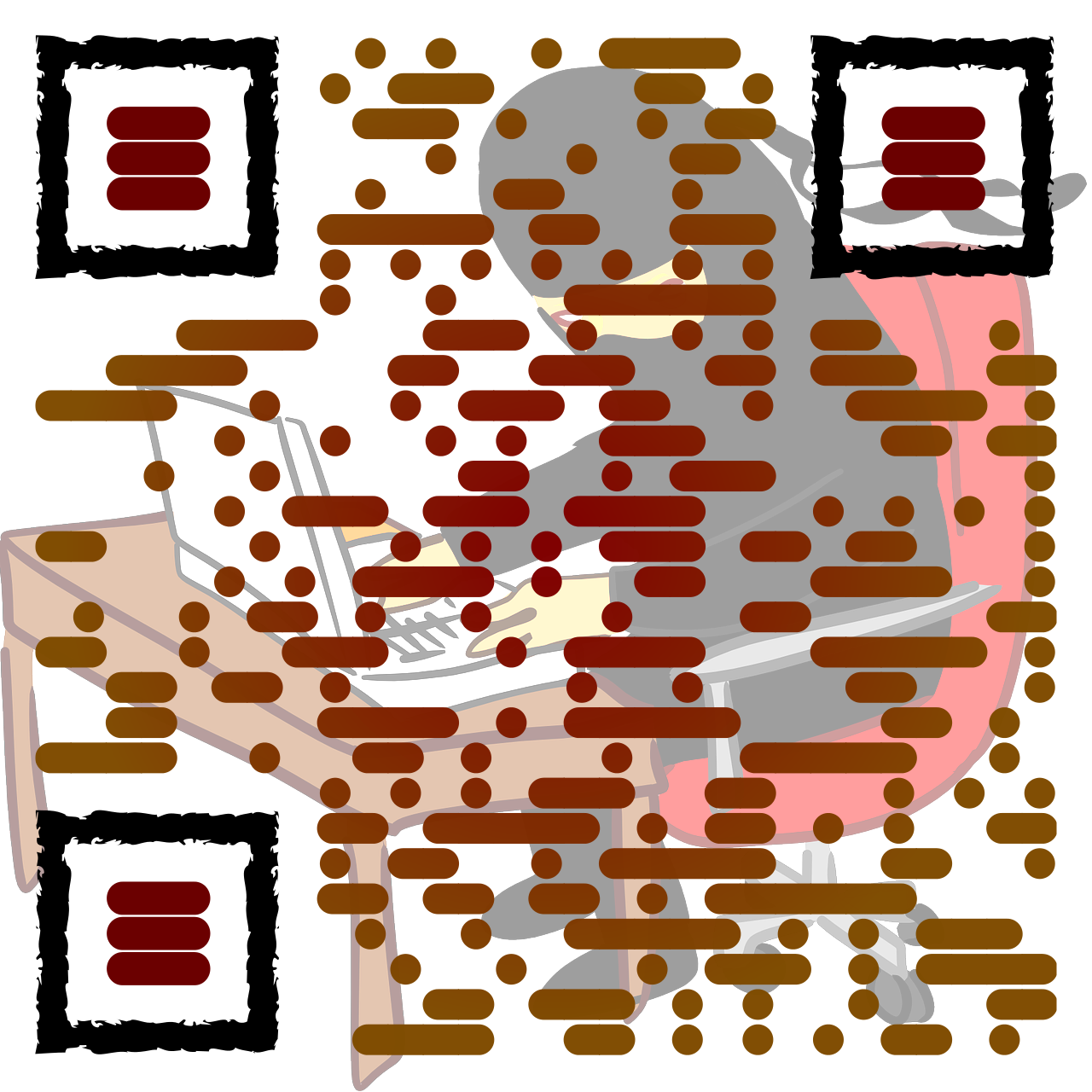

Supplement: S7 Fig — This Quick Response (QR) code, stylized to resemble a gapped multiple alignment of DNA sequences (including clip art from public domain at http://www.wpclipart.com), is a functional QR code provided for convenient linking to the NINJA-OPS source code and pre-built binaries hosted at http://ninja-ops.ninja, and for convenient sharing and distribution of the tool in poster presentations, slide shows, and other visual presentations. (TIF) [file pcbi.1004658.s007.tif]
